# Gallstone ileus with evident forchet sign:case report

**DOI:** 10.1016/j.ijscr.2019.06.063

**Published:** 2019-07-11

**Authors:** Nazmi Özer

**Affiliations:** Department of General Surgery, Adana City Training and Research Hospital, Adana, Turkey

**Keywords:** Gallstone ileus, Forchet sign, Rigler’s triad, Computerized tomography (CT), Enterolithotomy

## Abstract

**Introduction:**

Gallstone ileus (GSİ) is a rare complication of cholelithiasis (gallbladderstone), which may lead to obstruction of the small intestine. Particularly, computerized tomographic (CT) imaging method and special findings in these images help diagnosing of gallstone ileus. Treatment of this disease is surgery, surgery involves cholecystectomy + fistula repair + enterolitotomy, but it is controversial to perform cholecystectomy with enterolitotomy and fistula repair in the same session.

**Presentation of case:**

A 75-year-old male patient consulted to the emergency department with the complaints of nausea and vomiting. In the examinations of the patient, bilienteric fistula and gallstones that impacted in the jejunum leading to obstruction were observed in abdominal CT images of the patient who has ileus. The patient was evaluated as gallstone ileus. In addition, on tomographic images significant Forchet sign and Rigler’s triad images were viewed which were pathognomonic for gallstone ileus and did not have images as clear as in our case in the literature search. Laparotomy was performed on the patient due to the fact that he was elderly and the duration of anesthesia was wanted to be kept short and stone was extracted by enterolitotomy. Cholecystectomy and fistula repair were left for another session because of gallbladder and surrounding tissues were edematous. The patient was discharged with full recovery on the 6th post-operative day.

**Discussion–conclusion:**

As well as this disease is a rare cause of mechanical bowel obstruction, it is mostly seen in elderly patients. The most sensitive and specific imaging method in diagnosis is contrast-enhanced abdominal computerized tomography. In the tomographic images, especially the Rigler’s triad, Forchet sign and Petren sign are pathognomonic for gallstone ileus.

## Highlights

• GSI is clinical entity of mechanical intestinal obstruction.
• It’s most commonly seen in elderly patients.
• CT scanning is the most sensitive and spesific radiological diagnostic method. Also riglers triad, forchet sign and petren sign are pathognomonic for CT scanning in GSI.

## 1 Introduction

This work has been reported in line with the SCARE criteria [1].

Ileus is a clinical entity that is frequently encountered in our daily surgical practice due to many different causes. Gallstone is a rare cause of ileus which causes mechanical bowel obstruction [2]. Gallstone ileus is usually seen in elderly patients [3], the rate of ileus in the etiology is found as 1–4% [4,5] Female / male ratio is 3.5-6: 1; it is seen

more often in women. When the complications of gallbladder stone are evaluated in it self, the rate of causing ileus is seen as 0.3-0.5% [6].

In such cases, the state of inflammation (cholecystitis) due to the gallstone in gallbladder causes bilioenteric fistula by eradicating the gallbladder wall and adjacent gastrointestinal lumen. The gallstone passes into the small intestine from this fistula tract and causes obstruction in the small intestine or colon. The treatment of this disease is surgical intervention. Surgical intervention is planned as; enterolithotomy (removal of the stone) + cholecystectomy (removal of the gallbladder) + fistula repair. However, doing cholecystectomy and fistula repair in the same session during the enterolithotomy is a controversial issue, and many surgeons leave the cholecystectomy and fistula repair to the second operation.

Clinical, operational and radiological findings are evaluated together for the diagnosis of gallstone ileus, especially in some of the radiological findings which were taken after oral contrast enhancement in the computerized tomography, the ‘snake head like’ appearance known as the Forchet sign, which is seen as a result of the obstruction of the bowel lumen due to the gallbladder, being unable of the contrast agent to pass the stone and accumulating there, is pathognomonic in the diagnosis of the disease [6]. Also we will present a case of gallstone ileus, which appears to have a prominent forchet sign and that we think we will contribute to the literature, especially with images.

## 2 Case presentation

A 75-year-old male patient was admitted to the emergency service with the complaints of a 7-day history of colic-style abdominal pain, nausea and biliary vomiting. The patient whose vital signs were stable at the time of admission was.

In laboratory tests, C-reactive protein (CRP): increased as 10 mg / dl, the value of WBC (White blood count) was found as 16,000 μ/L high, and in biochemistry values glucose value was viewed as elevated by 414 mg/dL. There was no significant change in other tests.

**Fig. 1:**
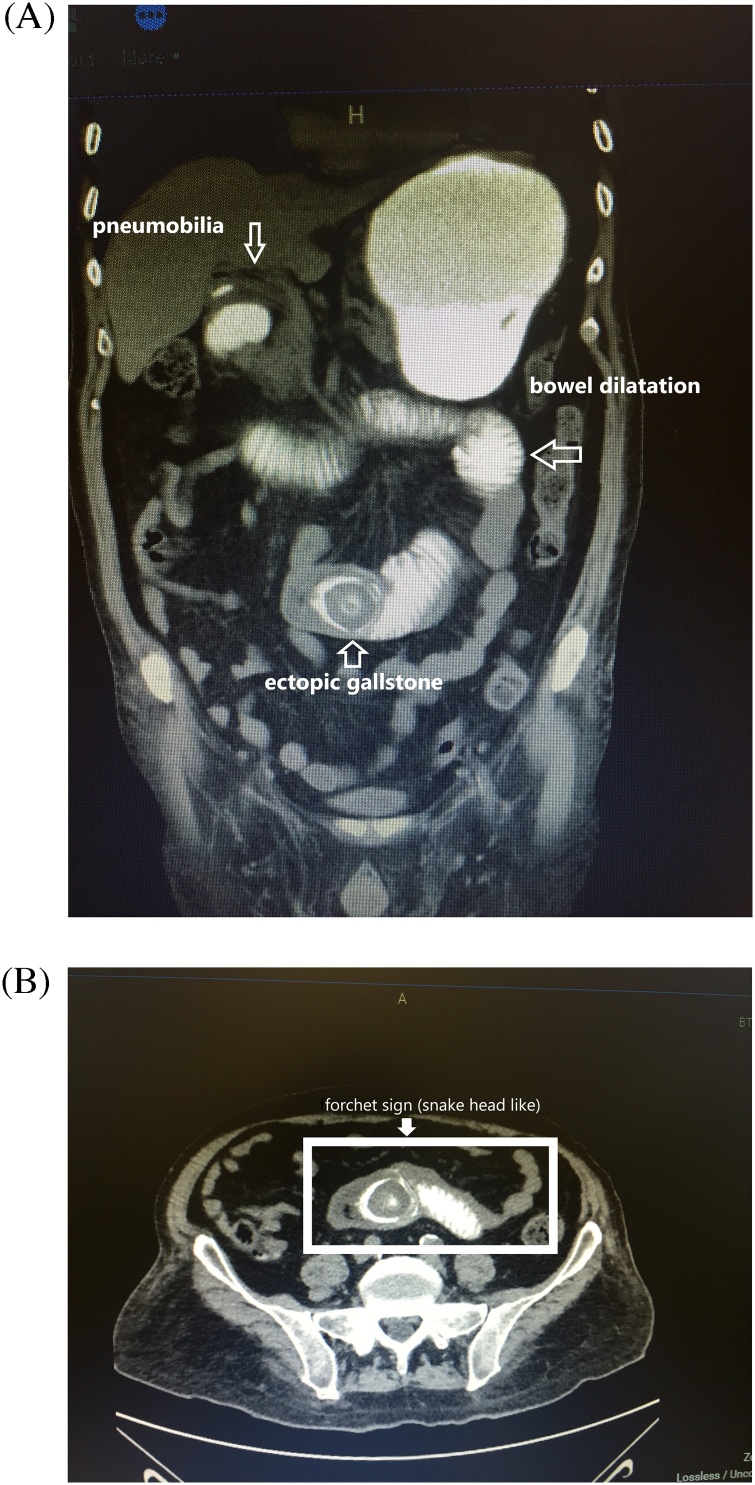
a – **Rigler’s triad** (ectopic gallstone, bowel dilatation, pneumobilia in biliary tract). b – Evident **Forchet sign** (snake head like).

Radiological examinations were performed and the patient was diagnosed as ileus. On the images of the patient who was performed oral-iv contrast-enhanced abdominal but, it was found that the patient had gallstone ileus after the Rigler’s triad and a prominently viewed forchet sign were seen (Fig. 1).

**Fig. 2:**
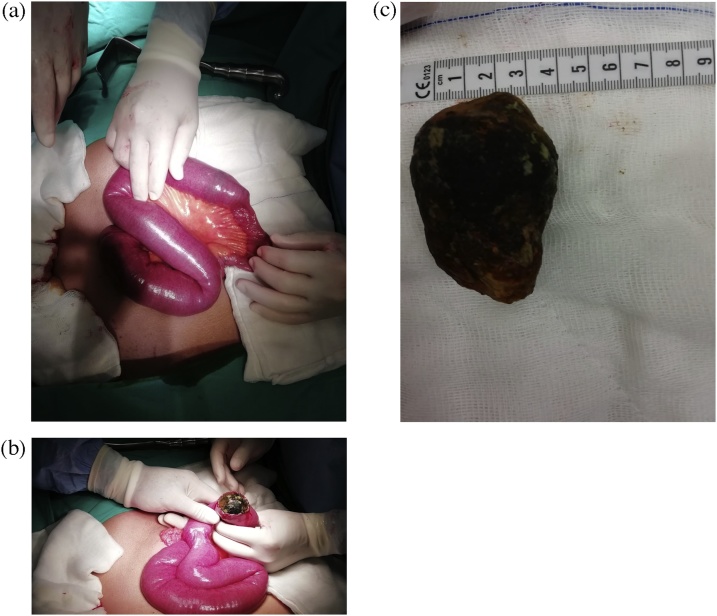
a – Jejenum completely occluded by a large mass. b – The mass extracted by enterolithotomy (gallstone). c – Size of gallstone (6 × 4 cm).

The patient was taken to the surgical intensive care unit due to her advanced age and dehydration, also having additional disease. After hydration he was operated on the 2nd day of his hospitalization. The operation was performed openly due to the fact that the duration of anesthesia was wanted to be kept short. In the exploration, it was seen that the segment of jejunum which was 80 cm distant from the treitz ligament was totally obstructed with a limited mobile, large mass. Intestinal segment of the mass in the proximal was seen widely. This mass lesion was initially predicted to be a bezoar, but after enterotomy it was found to be a 4 × 6 cm large gallstone. After enterolithotomy, small bowel was repaired double transversely (Fig. 2). When exploration was continued, it was seen that gallbladder was edematous and also there was cholecystoduodenal fistula between gallbladder and duodenum. Cholecystectomy and fistula repair were left for the second session because of the edematous tissues. The operation was terminated by placing a drain into the abdomen. The operation lasted a total of 80 min.

Antibiotic treatment was started to the patient before the operation and the treatment was completed in 48 h. The naso gastric catheter, which was inserted for aspiration and decompression, was withdrawn on the second post-operative day because the patient had gas outlet. At the postoperative 5th day, the abdomen drain was withdrawn from the patient who took diet of Regime 1 and tolerated. The patient was discharged with full recovery on the 6th post-operative day.

## 3 Discussion

In the gallstone ileus, the pathology is characterized by the formation of adhesions between the gallbladder and the gastrointestinal tract after high inflammation occurring during the recurrent attacks of acute cholecystitis, the increase of the pressure inside the lumen and secondarily to this the formation of bilioenteric fistulas as a result of occurring ischemia [3]. Then the gallstones pass from the bladder lumen to the small intestine from this formed fistula tract [4].

This pathophysiological mechanism resembles mirizzi syndrome. While tread is seen between the gallbladder and choledochus in mirizzi syndrome, on the other hand the pathology between the gallbladder and the gastrointestinal tract is monitored here [7].

The fistula occurring in gallstone is most commonly seen in duodenum (60–68%) [8], the other fistula regions can be seen as cholecystocolonic (5–25%), cholecysto-duodeno-colonic (2.5%) and less frequently cholecystojejunal, cholecystogastric fistulas.

The most impacting site of the stone is seen as the distal or terminal ileum [9] (75%). Also it can be located less frequently in duodenum (bouveretsyndrome), stomach, proximal ileum, jejunum and colon.

Gallstone ileus is divided into 3 groups, I – Acute classic gallstone ileus, II – Gallstone ileus with subacute recurrent attacks III – Chronic, gallstone ileus known as Kawersky syndrome with recurrent gallstones in the small bowel lumen [3]. We present a case of acute classical gallstone ileus.

However gallstone ileus is rare in the general population, it is responsible for 25% of small intestinal obstruction in the population over 65 years of age, it is also seen in female population more frequently than male population [10]. Our case was a 75-year-old male patient.

Preoperative diagnosis is difficult, but 40–70% of patients can be diagnosed. In the radiological examinations, only small bowel obstruction can be seen frequently. The Rigler’s triad consisting of images of pneumobilia (air in the biliary tract), ectopic gallstones, dilated small bowel loops is pathognomonic for this disease but these findings can not be seen in every cases.

In addition, the ‘snake head like’ appearance known as the **Forchet sign**, which is seen as a result of the obstruction of the bowel lumen due to the gallbladder, being unable of the contrast agent to pass the stone and accumulating there and **Petren sign** which is transfection of the contrast agent from the fistula tract to the gallbladder after giving oral contrast are also important radiological images for gallstone ileus [3].

In the diagnosis of gallbladder stones, the sensitivity, specificity and accuracy of CT were reported as 93%, 100% and 99% respectively [11,12]. Rigler’s triad is seen more clearly and apparently in computed tomography sections than in x-ray films [13,14]. Also in our case, the Rigler’s triad and Forchet sign are clearly seen in tomographic images.

The main treatment in gallstone ileus is surgery. Surgical procedure is performed in 3 separate ways 1 – Simple enterolithotomy + primary bowel repair 2 – Single stage treatment procedure, enterolithotomy + cholecystectomy + fistula repair 3 – Double stage treatment procedure, first enterolithotomy, in the second stage cholecystectomy + fistula repair is considered [2,3]. Here, the choice of surgical procedure is a controversial issue. There are some opinions towards cholecystectomy and fistula repair in the second session after enterolithotomy + primary bowel repair will increase mortality due to the patient’s advanced age and comorbid diseases [[11], [12], [13]]. In our case, we preferred enterolithotomy + primarybowel repair because our patient had advanced age and comorbid diseases as well as gallbladder and surrounding tissues were edematous.

## 4 Conclusion

Gallstone ileus; is one of the factors involved in the etiology of intestinal obstruction, it is most commonly seen in elderly patients, CT scan is the most sensitive and specific radiological diagnosing method for gallstone ileus, especially Rigler’s triad, Forchet sign and Petren sign are pathognomonic.

## Funding

The authors declared that this study has received no financial support.

## Ethical approval

This case report exempted from ethical approval.

## Consent

We have a confirmation with patient for this case report.

## Author contributions

Concept – N.Ö.; Design – N.Ö.; Supervision – N.Ö.; Data Collection and/or Processing –N.Ö.; Analysis and/or Interpretation – N.Ö.; Literature Search – N.Ö.; Writing Manuscript – N.Ö.; Critical Reviews – N.Ö.

## Registration of research studies

NÖ.

## Guarantor

Nazmi Özer is guarantor for this work.

## Provenance and peer review

Not commissioned, externally peer-reviewed.

## Declaration of Competing Interest

The authors have no conflict of interest to declare.
